# Strategies to optimize modeling habitat suitability of *Bertholletia excelsa* in the Pan‐Amazonia

**DOI:** 10.1002/ece3.5726

**Published:** 2019-10-25

**Authors:** Daiana C. M. Tourne, Maria V. R. Ballester, Patrick M. A. James, Lucieta G. Martorano, Marcelino Carneiro Guedes, Evert Thomas

**Affiliations:** ^1^ Environmental Analysis and Geoprocessing Laboratory CENA University of Sao Paulo Sao Paulo Brazil; ^2^ Department of Biological Sciences University of Montréal Montreal QC Canada; ^3^ Agrometeorology Laboratory EMBRAPA Eastern Amazon Santarem Brazil; ^4^ Forest Research and Development EMBRAPA Amapá Macapá Brazil; ^5^ Bioversity International Regional Office for the Americas Lima Peru

**Keywords:** Amazon‐nut, Brazil‐nut, expert knowledge, maximum entropy, model evaluation, principal component analysis, protected Amazonian species, spatial filtering, species distribution model

## Abstract

**Aim:**

Amazon‐nut (*Bertholletia excelsa*) is a hyperdominant and protected tree species, playing a keystone role in nutrient cycling and ecosystem service provision in Amazonia. Our main goal was to develop a robust habitat suitability model of Amazon‐nut and to identify the most important predictor variables to support conservation and tree planting decisions.

**Localization:**

Amazon region, South America.

**Methods:**

We collected 3,325 unique Amazon‐nut records and assembled >100 spatial predictor variables organized across climatic, edaphic, and geophysical categories. We compared suitability models using variables (a) selected through statistical techniques; (b) recommended by experts; and (c) integrating both approaches (a and b). We applied different spatial filtering scenarios to reduce overfitting. We additionally fine‐tuned MAXENT settings to our data. The best model was selected through quantitative and qualitative assessments.

**Results:**

Principal component analysis based on expert recommendations was the most appropriate method for predictor selection. Elevation, coarse soil fragments, clay, slope, and annual potential evapotranspiration were the most important predictors. Their relative contribution to the best model amounted to 75%. Filtering of the presences within a radius of 10 km displayed lowest overfitting, a satisfactory omission rate and the most symmetric distribution curve. Our findings suggest that under current environmental conditions, suitable habitat for Amazon‐nut is found across 2.3 million km^2^, that is, 32% of the Amazon Biome.

**Main conclusion:**

The combination of statistical techniques with expert knowledge improved the quality of our suitability model. Topographic and soil variables were the most important predictors. The combination of predictor variable selection, fine‐tuning of model parameters and spatial filtering was critical for the construction of a reliable habitat suitability model.

## Graphical abstract

Innovation on fit models using collaborative model‐building process. Ecological knowledge supporting modeling techniques. Suitability habitat model to support conservation and planting to important Amazon species.

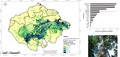

## 1 INTRODUCTION

Range‐wide management and conservation of socio‐economically important tree species require a comprehensive understanding of species habitat preferences and the magnitude and nature of anthropogenic and natural threats to their in situ persistence. However, in Amazonia such knowledge often remains incomplete and based on local experience rather than rigorous scientific data. Furthermore, existing knowledge on Amazonian forest species has been poorly integrated within conservation planning frameworks (Addison et al., 2013; Gardner, Barlow, Chazdon, Robert, & Harvey, 2009). As a result, biodiversity conservation strategies have failed to protect the majority of endemic species in Brazil (Oliveira et al., 2017). The latter authors found that <40% of the estimated distribution area for some species fell inside protected areas. Conservation decision‐making processes in Amazonia can be greatly improved through the inclusion of species distribution models (SDMs) which is currently not the case in most Amazon countries.

The complexity of SDMs, both in terms of development and understanding, is a common constraint to their application in decision‐making (Addison et al., 2013). Nonetheless, SDMs are an increasingly important tool for predicting habitat suitability and for understanding species environmental tolerances (Stolar & Nielsen, 2014). SDMs are also essential to guide field collections, as well as to inform or reinforce management, reforestation, and conservation plans (Franklin, 2010). The value and importance of a well‐constructed SDM have motivated an explosion of methods aimed at building more accurate models (Elith et al., 2011; Kuhnert, Martin, & Griffiths, 2010). However, few efforts have been made to develop a collaborative model‐building process among modelers, ecologists, and decision‐makers to improve model quality (Calixto‐Pérez et al., 2018) and to facilitate clear communication of model results (Addison et al., 2013).

One of the most widely used methods of developing SDMs is MAXENT (Phillips, Anderson, Dudík, Schapire, & Blair, 2017). MAXENT is a correlative model based on the principle of maximum entropy to predict or infer species occurrence using presence‐only data and environmental variables (Phillips, Anderson, & Schapire, 2006). The probability of occurrence is then modeled using a logistic equation fitted to presence data and background locations chosen randomly or in target‐groups that MAXENT contrasts against the presence (Phillips & Dudík, 2008). Several studies have highlighted that the performance of MAXENT models is influenced by (a) biases in occurrence data that cause overfitting (Kramer‐Schadt et al., 2013) and (b) the uncritical use of default settings based on taxonomic groups studied by MAXENT designers (Phillips & Dudík, 2008). Indeed, there is growing evidence that the most appropriate settings vary according to species and study area. However, only 3.7% of articles published between 2013 and 2015 tested if the default regularization and feature class parameters were appropriate for their data (Morales, Fernández, & Baca‐González, 2017; Radosavljevic & Anderson, 2014). When adequately fine‐tuned, these parameters prevent the algorithm from fitting the input data too closely (Phillips & Dudík, 2008).

Errors can furthermore be introduced into MAXENT‐based analysis through multi‐collinearity among predictors that can inflate the variance and standard errors of regression parameter estimates. Careful selection of candidate predictor variables is therefore recommended (Dormann et al., 2013). Statistical analysis has been commonly used to address this issue, as for example through principal component analysis (PCA) (Everitt & Dunn, 2001). However, models using maximum entropy have also been improved by integrating expert knowledge in the predictor selection stage of model development (Porfirio et al., 2014).

An expert is someone who has gained knowledge through his/her life experience, education, or training, and who is responsible for providing judgments (Mcbride & Burgman, 2012). Experts can contribute, for example, to the choice of variables based on their knowledge of a species' life cycle (Porfirio et al., 2014), to determine geographic limits to the presumed species (Jones, Dye, Pinnegar, Warren, & Cheung, 2012), to provide knowledge when empirical data are lacking (Kuhnert et al., 2010), or simply to provide feedback on model results. Expert‐based information has been successfully used to improve management of environmental systems (Perera, Drew, & Johnson, 2012), but has been seldom used in the development of SDMs (Kuhnert et al., 2010; Porfirio et al., 2014).

### 1.1 Amazon‐nut modeling distribution

MAXENT has been applied previously to model the distribution of the Amazon‐nut (*Bertholletia excelsa*), specifically at Para State, Brazil (Albernaz & Avila‐Pires, 2009). However, results from this study are limited in their utility for conservation planning due to paucity of presences used, the restricted spatial extent of analysis, and the limited diversity of environmental predictors considered. Thomas, Alcázar, Loo, and Kindt (2014) also examined Amazon‐nut distribution using an ensemble modeling approach. Their goal was to assess the distribution of Amazon‐nut across the Amazon basin and make projections to past and future climate conditions. They found that the current spatial distribution of this species was shaped by an initial period of range contraction in the Pleistocene, followed by range expansion in the Holocene resulting in its contemporary distribution. Although these findings are informative and compelling, the model of distribution of suitable habitat estimated showed a high degree of overfitting and had limited out of sample (OOS) predictive power. Such reduced predictive power was clearly observed at eastern Amazon, where they had few records of presence.

Developing robust models with a high OOS for the Amazon‐nut is now possible thanks to the availability of high‐quality environmental data (e.g., Wordclim (Fick & Hijmans, 2017) and Soilgrid platforms (Hengl et al., 2014)). Additionally, species occurrence data are being generated in scientific collaboration networks with standardized accessibility policies (e.g., Global Biodiversity Information Facility; https://www.gbif.org). Specifically for *B. excelsa*, a Brazilian project named MAPCAST “Mapping of Amazon‐nut groves and socio‐environmental and economic characterization of Amazon‐nut production systems in the Amazon” was carried out from 2013 to 2018 (https://www.embrapa.br/en/projetos). It generated direct biological information and the formation of a group of specialists on this taxonomic group.

Amazon‐nut (*Bertholletia excelsa*) is one of the largest and longest living hyperdominant tree species in Amazonia (Ter Steege et al., 2013). In old‐growth forest, Amazon‐nut trees can reach 60 m in height and 4 m in diameter at breast height (Müller, 1995). It ranked third in the top 20 accumulators of aboveground woody biomass in Amazonia (Fauset et al., 2015) and has provided critical ecosystem services to humans since prehistory (Roosevelt et al., 1996). The fruits are hard, indehiscent, and can often be opened by large species of psittacid *(Ara* sp) and rodents (*Agouts* sp). Agouts and human both play an important role in Amazon‐nut dispersion, rodents can carry fruits as far as 60 m (Haugaasen, Haugaasen, Peres, Gribel, & Wegge, 2012) and humans positively influenced its abundance in the past (Thomas et al., 2014). Species distribution differs for being broad and discontinuous, leading to formation of groves in some areas (Salomão, 2009) and scattered trees in others (Wadt, Kainer, & Gomes‐Silva, 2005). However, its trees population has been vulnerable to illegal activities in the Amazon for the last forty years, mainly in southern and eastern Amazon, region named “arch of deforestation” (Scoles, Canto, Almeida, & Vieira, 2016).

Amazon‐nut is legally protected and one of the most important nontimber forest product (NTFP), on which tens of thousands of local people depend, mainly in Brazil, Bolivia, and Peru (Guariguata, Cronkleton, Duchelle, & Zuidema, 2017). The fruit's success is recently attributed to health benefits offered by the seeds rich in selenium and other micronutrients (Cardoso, Duarte, Reis, & Cozzolino, 2017). Its cultural and economic importance brings various common names constantly associated with geographic localization (Brazil‐nut, Pará‐nut, Acre‐Nut, and Bolivian Brazil‐nut) to the fruit market. Here, we adopted the term Amazon‐nut instead of Brazil‐nut, the most common name, to be more inclusive of other Amazonian countries, in which the species is native. Extensive research has been dedicated to evaluating the sustainability of nut harvesting (Bertwell, Kainer, Cropper, Staudhammer, & Oliveira Wadt, 2017), characterizing demographic and genetic structure within and among (Salomão, 2009; Sujii, Martins, Wadt, Azevedo, & Solferini, 2015), and understanding the natural and human drivers of its current distribution (Thomas, Alcázar Caicedo, Mcmichael, Corvera, & Loo, 2015). Despite this rich body of work, surprisingly little is known about the environmental predictors that determine species occurrence.

In this paper, we develop a novel SDM using MAXENT with the goal of improving our understanding of the habitat extent and the suitable environmental to *B. excelsa* occurrence, in order to guide conservation and tree planting strategies. Given the importance of careful selection of potential predictor variables and removal of bias in SDMs (Boria, Olson, Goodman, & Anderson, 2014; Franklin, 2010), we also address three methodological questions: (a) Which strategy of predictor selection is most adequate to model *B. excelsa* habitat suitability? (b) What are the best MAXENT settings based on the distribution of our data across Amazonia? (c) What is the minimum distance between occurrence points to remove bias and fit robust models statistically and ecologically? Finally, we evaluated the usefulness of incorporating expert knowledge in predictor variable selection for enhancing the quality of SDM for *B. excelsa*.

## 2 METHODS

### 2.1 Occurrence data

**Figure 1:**
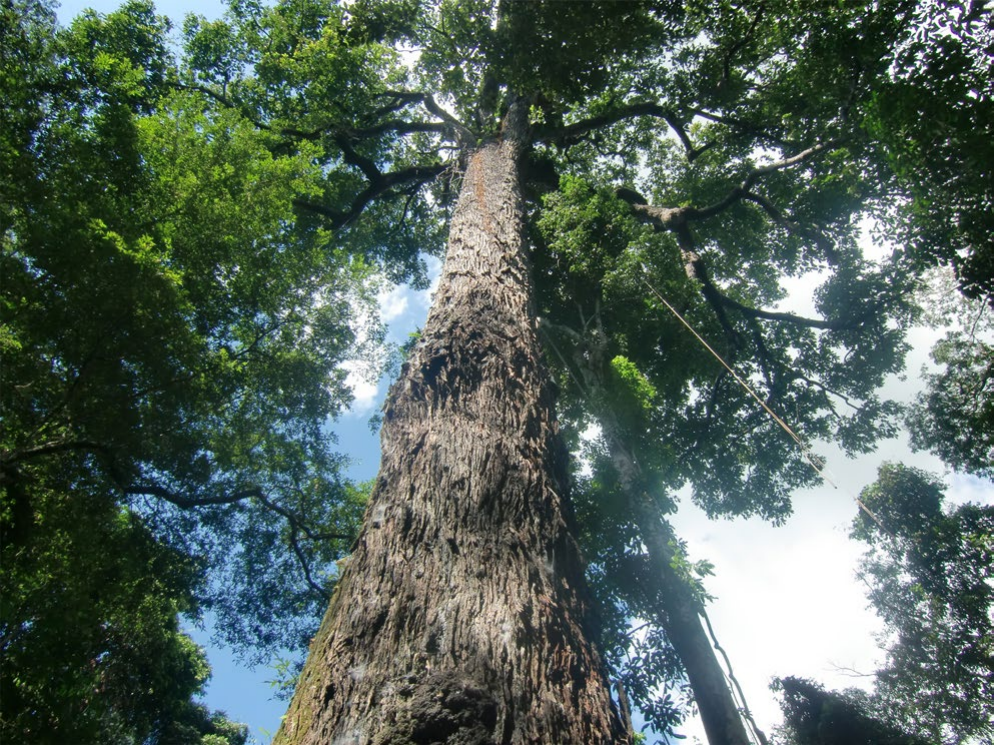
Amazon‐nut (*B. excelsa*) tree with 10.65 m of circunference mesured at breast height in 2016. It was found in a forest fragment of the rural agroextractivist settlement Praia Alta Piranheira, in Nova Ipixuna do Pará, Brazil. This specimen is known as “majestade” (Majesty) in this rural community

**Figure 2:**
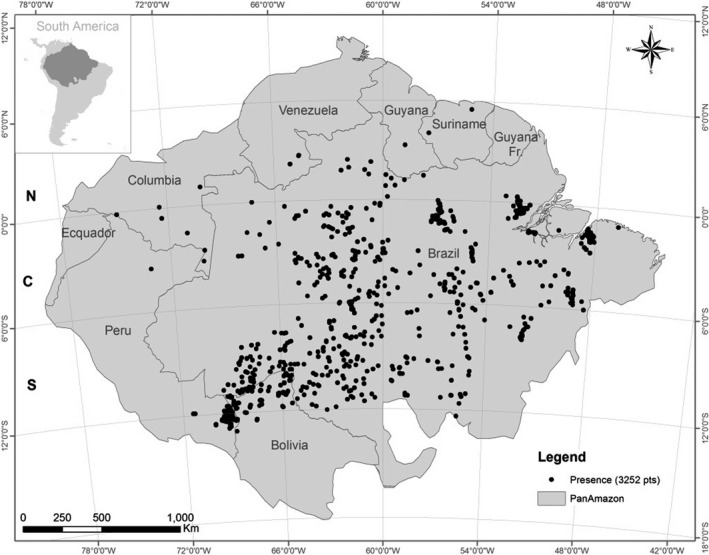
Geographical localization of the Amazon in South America. The black points indicate the localization of the Amazon‐nut (*B. excelsa*) observation points obtained to this study (3,252). The coordinate system adopted was Albers equal‐area conic projected for continental areas

This study was conducted in the Amazonia biome, the world's largest tropical rainforest, occupying 7.2 million of km^2^. Amazon‐nut's occurrence data (*n* = 3,325) were collected from a diversity of sources: datasets provided by researchers acquired in field collection; data available from Emilio Goeldi Museum and Embrapa herbarium collections; Global Biodiversity Information Facility (GBIF) database; scientific publications and data recorded in field expeditions from 2015 to 2018 supported by São Paulo Research Foundation (see Table S1.1). In Figure 1, we exhibited the biggest specimen tree found in our field expedition in 2016, and in Figure 2, the spatial distribution of the presence data obtained.

### 2.2 Environmental data

Predictor variables were derived from globally available raster data at 30 arc‐second spatial resolution (~1 km). A total of 102 predictors were assembled for this study (Table S1.2). Nineteen bioclimatic variables were obtained from http://www.worldclim.org, which are based on interpolation data from 1950 to 2000 (Hijmans, Cameron, Parra, Jones, & Jarvis, 2005). From these layers, monthly potential evapotranspiration (PET), aridity (ARI), and soil water content (SWC) layers were calculated and available by http://www.cgiar-csi.org/data.

Predictors for physical and chemical soil properties were obtained for seven depths (0 up to 200 cm) from https://www.soilgrids.org. Selected predictors included soil organic carbon (g/kg), soil pH × 10 in H_2_O, sand, silt and clay fractions (%), bulk density (kg/m^3^), cation‐exchange capacity (cmol^+^/kg), and coarse fragments (%) (Hengl et al., 2014). To test for differences between soil variable means at different depths, we used ANOVA followed by post hoc *Tukey tests* computed using the *multcompView* package for R (Graves, Piepho, Sundar, Maintainer, & Selzer, 2015). Prior to ANOVA, we verified the assumption of normality and homogeneity of data variance, using *Shapiro–Wilk* and *F tests*, respectively. To minimize multi‐collinearity among soil predictors, only layers with significantly different mean values were retained for further modeling. Boxplots are given in Figure S1.1.

Global terrain elevation data (GMTED2010) were retrieved from the USGS/ NASA database: https://topotools.cr.usgs.gov/gmted_viewer/. These data were used to derive topography and hydrological variables, such as slope, aspect, Compound Topographic Index, and Stream Power Index via ARCGIS 10.3. Geological data were also obtained from NASA https://daac.ornl.gov/SOILS/guides/Global_Soil_Regolith_Sediment.html. These variables provided estimates of the thickness of the permeable layers above bedrock like soil, regolith, and sedimentary deposit (Pelletier et al., 2016).

### 2.3 Removing bias

One source of inaccuracy in SDMs is sampling bias in presence data (Boria et al., 2014). For example, it has been shown that presences recorded may suffer from problems of locational under‐specification, geocoding errors, taxonomic changes, among others (Franklin, Serra‐Diaz, Syphard, & Regan, 2017). To minimize potential biases, we removed all presences within a radius of 5 km from municipal centers. For this, we created a distance surface from cities using Euclidian distance on geographic information system (GIS). Additionally, presences in savanna within the Amazon biome were not used because there were few Amazon‐nut occurrences found in these dry forest patches, and species persistence in these drier environments requires further investigation. The omission of samples from the Cerrado regions was also strongly suggested by experts consulted. After this first filtering, 3,252 occurrence records remained.

Bias also occurs when presence data are spatially clustered, often due to more frequent sampling in regions that are more accessible, or due to dispersal limitation causing natural clusters. Consequently, parts of the environmental space suitable for a species are overrepresented, while other parts are absent or poorly represented (Fourcade, Engler, Rödder, Secondi, & Brooks, 2014). Inconsistent spatial representation of potential species habitat can lead to overfitting (Radosavljevic & Anderson, 2014) and biased inference. To address this problem, spatial filtering can be used reducing overrepresentation and improving model quality (Boria et al., 2014; Kramer‐Schadt et al., 2013).

To reduce biases and find optimal geographic distance between trees, we filtered our Amazon‐nut presence data through random rarefication of 3,252 presences considering minimum Euclidian distances between them of 3, 5, 10, 15, and 20 km. Filtering was implemented using the *Sdmtoolbox* ArcGIS toolbox (Brown, 2014). These distances were selected because many of the presence points in our dataset were clustered at spatial resolutions below 1 km, the grain size of our predictor variables, in order to evaluate how environmental heterogeneity was maintained according to the increasing geographic distance. These experiments have been suggested in the literature (Boria et al., 2014).

### 2.4 Predictor selection

When developing SDMs, researchers often prioritize predictors associated with primary plant resources (e.g., water, temperature and nutrients) or those related to human or natural disturbance (e.g., fire and insect outbreaks) (Guisan & Thuiller, 2005). However, problems may arise on the one hand because predictors choices may be subjective and on the other because subsets of predictors may be highly correlated. Automated model selection methods have been developed to address some of these subjectivity issues, because they apply different combinations of variables without researcher's interference to find the “best” model. This selection is given by the model weight that is computed by means of metrics, such as minimization of the Akaike information criterion (Burnham & Anderson, 2002). Despite the advances, it remains challenging to identify a meaningful and informative subset of SDM predictors (Galipaud, Gillingham, David, & Dechaume‐Moncharmont, 2014), largely due to the confounding influence of multi‐collinearity (Dormann et al., 2013).

Principal component analysis (PCA) has been suggested to reduce the dimensionality of predictor variables through the generation of multiple orthogonal synthetic variables (Everitt & Dunn, 2001). The downside is that PCA results can be difficult to interpret, especially when trying to determine which variables contribute meaningfully to each component (Vaughan & Ormerod, 2005). However, also external information can be useful to interpretation of ecological structure. Expert knowledge previously considered to be subjective has recently been recognized for its vast potential to improve ecological models (Kuhnert et al., 2010; Porfirio et al., 2014). Here, we compare three methods for variable selection: one based on ordination (PCA), one based on expert knowledge, and another that combines both approaches.

#### 2.4.1 Ordination‐based variable selection

We used PCA to reduce collinearity and dimensionality in our large predictor dataset (Legendre & Legendre, 2012). PCA‐derived synthetic predictors were calculated using environmental data covering the entire geographic space of the Amazon (Pan‐Amazon) at spatial resolution of 30 arc seconds (~1 km). Prior to analysis, all predictors were standardized to zero mean and unit variance. Then, we explored correlations between variables using Pearson's R. We grouped variables by category (i.e., climatic, edaphic, and geophysical) and submitted each group to a PCA. The eigenvectors were normalized for each group, and we retained the subset of principal components accounting for 80% of the variance in the original data (Jolliffe, 1972).

The predictors that maximally contributed to explaining variance in principal components were identified based on correlations between variables and PCA axes (eigenvector and its standard deviation). Using graphics produced in the *factorextra* package for R (Kassambara & Mundt, 2017), we obtained the contribution of each variable to the overall axis expressed as a percentage. Only the variables whose contribution was greater than the average were retained. We iteratively recalculated a new PCA on this restricted set of predictors. Next, we applied collinearity tests at a 95% confidence interval using *mctest* package for R (Ullah & Aslam, 2017). Once collinearity persisted, one for every two variables with Pearson *R* > |.7| in the correlation matrix was rejected. The variables retained following this procedure will be referred to as Group 1.

#### 2.4.2 Expert‐based variable selection

In 2016, we convened an expert panel in Amapa State, Brazil, composed of twelve researchers (PhDs and graduate students) with different types of expertise on Amazon‐nut to a workshop titled “Maxent modelling and its application in the estimation of preferential areas to *B. excelsa*,” organized by the MAPCAST project. The panel was established to collect expert knowledge on the geophysical and biological factors influencing Amazon‐nut distribution for incorporation in the SDM building process.

The 102 variables in the original database, as well as preliminary PCA results, were submitted to their appraisal. The panel of experts was specifically asked the following questions: Which variables should be included in the model? What is the maximal period during which the plant can be exposed to water and heat stress? Should all available depths of soil variables be used? Were the variables selected by PCA adequate to model *B. excelsa*? After analysis and discussion, the experts reached consensus and provided a list of what they considered to be the most important variables for the occurrence of Amazon‐nut (Table S1.2). This variable set will be referred to as Group 2. Combining both approaches, we also calculated a PCA from the set of variables selected by the expert panel. The variable set will be referred to as Group 3.

### 2.5 Setting and fitting models

We calibrated and projected all models using the *ENMeval* package for R (v. 0.2.2; Muscarella et al., 2014), which includes some of the latest functions developed to help modelers find parsimonious models using maximum entropy (v. 3.3.3k; Phillips et al., 2017).

Presence data were used to determine appropriate feature classes (FC) and regularization multiplier (*β*) parameters within MAXENT for our study area. Feature classes are functions (linear, quadratic, hinge, product, threshold, and categorical) created by MAXENT for each environmental variable (Phillips & Dudík, 2008). By default, features choice is usually conditioned by the number of observations (*n*). When *n* > 80, all features are used and consequently, model complexity increases (Elith et al., 2011). To reduce complexity, users can specify FCs manually and adjust the level of regularization via the multiplier coefficient (*β*), which controls the smoothness of the distribution curve. It is equilibrated by lambda regularization parameter in the regression equation. By default, *β* = 1 is often selected to balance fit and complexity, but studies have mentioned that higher values result in smoother models (Elith et al., 2011), while according to others, values of *β* above 4.0 may lead to decline in models quality (Radosavljevic & Anderson, 2014).

We sought to identify the best FC and *β* parameters for our MAXENT model of Amazon‐nut occurrence. As such, we examined five feature classes and combination thereof (L, H, T, LQ, LQP, LQH, LQHP, LQHPT, where L = linear, Q = quadratic, H = hinge, P = product, and T = threshold), and four levels of regularization from 0.5 to 2.0, in increments of 0.5. We examined the suitability of these combinations of parameters for both filtered (rarefied occurrence) and unfiltered models for each of the three groups of candidate predictors. We used random *k*‐fold cross‐validation selection of training and testing data, adopting *k* = 10 to assess model accuracy (Kohavi, 1995). Overall, 576 models were run, taking 10,000 random pseudo‐absences from the Pan‐Amazonia background (Phillips & Dudík, 2008).

### 2.6 Model performance

Model performance was evaluated using the three metrics: (a) the corrected Akaike information criterion (AICc) (Burnham & Anderson, 2002); (b) the area under the curve of the receiver operating characteristic (ROC) for the test data (AUCTEST) (Elith et al., 2011; James, Robert, Wotton, Martell, & Fleming, 2017); and (c) the 10% training omission rate (OR10) (Fielding & Bell, 1997; Liu, White, & Newell, 2013). All metrics were calculated using the *ENMeval* package in R (Muscarella et al., 2014).

We compared all models with ΔAICc < 2, which indicates equivalent models (Burnham & Anderson, 2002) using AUC and OR10 values to identify the most appropriate groups of predictors and filtering distance. Even based on these three metrics, it was not trivial to select the most appropriate spatial filtering distance. For this reason, we ran MAXENT using R *dismo* package just for the six best models (Hijmans, Phillips, Leathwick, & Elith, 2011). This resource was chosen because it offers some useful functions to complement our model evaluation, as *nicheOverlap* and *evaluate*.

We used the *nicheOverlap* function to compute Schoener's D statistic (Warren, Glor, & Turelli, 2008), which quantifies pairwise similarities among the best unfiltered and filtered models. Confusion matrices were also reevaluated using the *evaluate* function for constructing density curves, and determining the relative contributions of environmental variables, as well as different thresholds. Continuous maps were transformed into binary maps using the maximum sensitivity and specificity sum (max SSS threshold). This threshold has provided good results when reliable absence data are unavailable (Liu et al., 2013). Pixels with values equal to or higher than the threshold were considered suitable.

**Figure 3:**
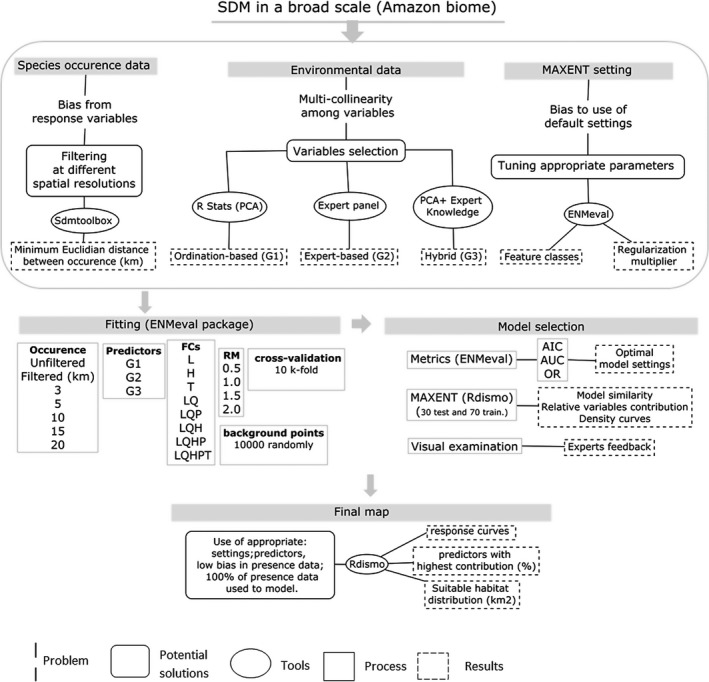
Summary of the model‐building process executed to identify the suitable habitat for Amazon‐nut (*B. excelsa*) in the Pan‐Amazon

The final maps were examined visually by six of twelve Amazon‐nut experts consulted who were asked to provide feedback on three aspects: (a) whether the model showed predictive power to identify underrepresented areas; (b) whether the distribution of the habitat of the *B. excelsa* had been well‐represented; (c) whether the most important selected variables made ecological sense. This information was used in complement to the statistical metrics. The model‐building process is summarized in Figure 3.

## 3 RESULTS

### 3.1 Candidate predictor variables

**Figure 4:**
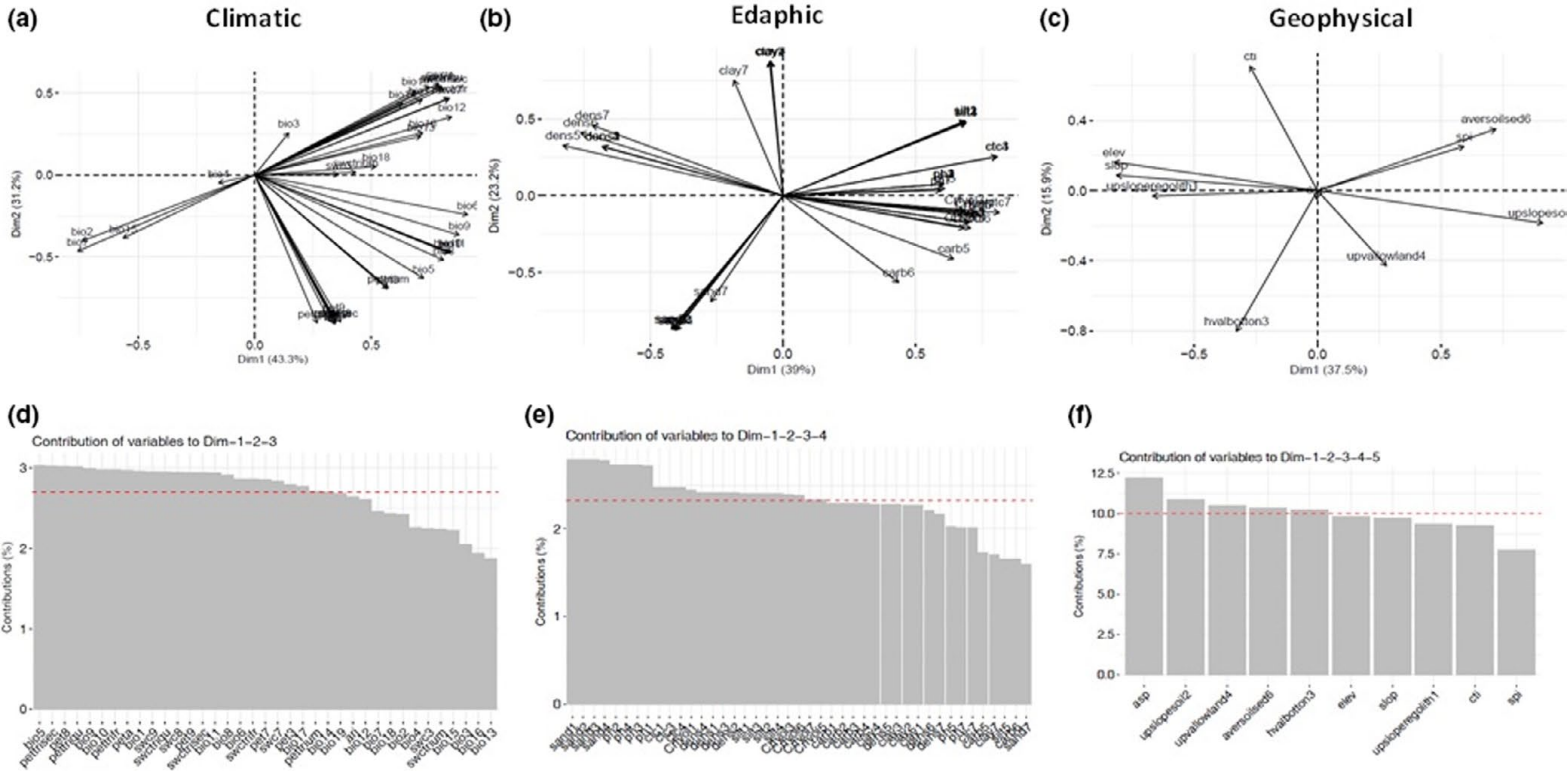
The first two principal axes of PCA for the environmental predictors in the Amazon geographical space (Group 1): (a) 37 climate variables; (b) 43 soil variables; and (c) 10 geophysical variables. Variables percentage of contribution in the principal components with the large variance of the data. The red‐dashed line indicates the expected average contribution: (d) 22 climate variables; (e) 24 soil variables; and (f) 5 geophysical variables were selected

For Group 1, we identified moderate to high correlations among predictor variables within the climatic (Figure 4a), edaphic (Figure 4b), and geophysical (Figure 4c) predictor groups. For the set of 37 analyzed climate variables, 87% of the variance was explained by the first three ordination axes. For the soil (*n* = 43) and geophysical (*n* = 10) predictors, four and five axes, respectively, were required to capture at least 80% of the variance.

Following initial examination of PCA results, we retained 22 climate variables, 24 soil variables, and five geophysical variables which have loadings on the respective PCA axes greater than average (Figure 3d–f). These variables were again submitted to a PCA which resulted in a new set of PCA scores by category. From this PCA 17 climatic variables, 16 soil variables and three geophysical variables had the highest contributions. Several of these variables remained correlated. To reduce multi‐collinearity, we retained only variables with pairwise correlations (Pearson's *r*) <.7. Temperature of the driest quarter and evapotranspiration of driest quarter had a stronger relationship with the first axis, whereas soil water content of driest quarter had a stronger correlation with the second one. The soil and geophysical variables that were most correlated with the first three axes were bulk density (fine earth) in kg/m^3^, soil pH‐H2O, silt mass fraction %, aspect, hillslope valley‐bottom and average soil, and sedimentary deposit thickness. Additional details on ordination including factor loadings can be found in Table S2.1.

For Group 2, 29 environmental variables of the initial set of 102 were highlighted by experts (Table S1.2). They included only two soil depths, one superficial (0–5 cm) and the other deeper (100–200 cm), to represent variation of the soil variables. Among the climatic variables, temperature and soil water content of the driest quarter were indicated to represent stressful periods, as well as, annual precipitation because water supply is a determining factor for fruit production.

**Figure 5:**
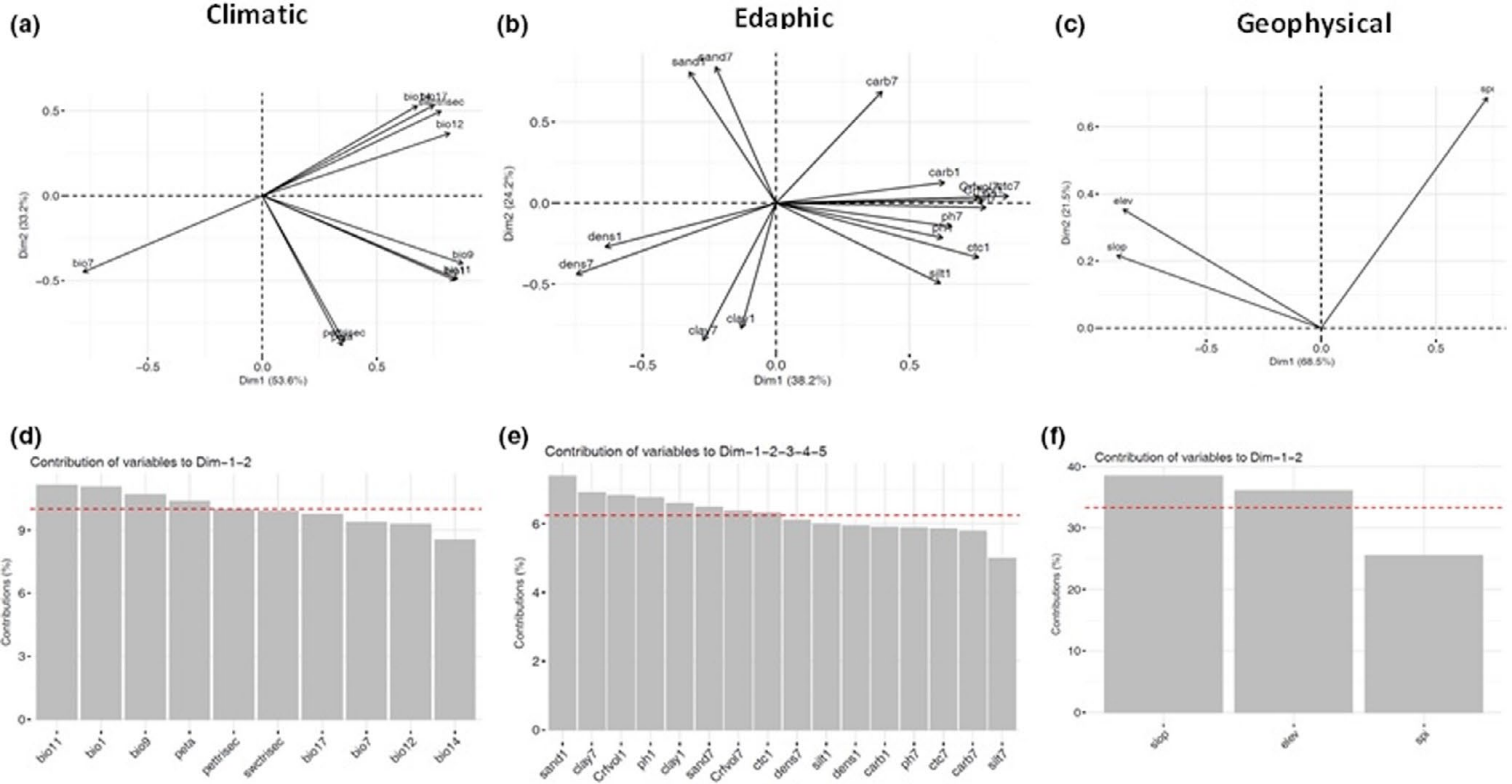
The first two principal axes of PCA for the environmental predictors proposed by experts in the Amazon geographical space (Group 3): (a) 10 climate variables; (b) 17 soil variables; and (c) 3 geophysical variables. Variables percentage of contribution in the principal components with the large variance of the data. The red‐dashed line indicates the expected average contribution: (d) 4 climate variables; (e) 8 soil variables; and (f) 2 geophysical variables were selected

A PCA based on the variables selected by experts (Group 3) captured most of the variance in the first two ordination axes of climatic (86.8%) and geophysical (90.0%) predictors. Four climatic and two geophysical variables showed contributions above average: Mean temperature of driest quarter showed the highest correlation with the first axis, followed by mean temperature of the coldest quarter and annual mean temperature, whereas the annual potential evapotranspiration had stronger relation with the second axis. The relation between the predictor variables and the first two principal components are visualized in Figure 5a–c.

Among the geophysical predictors, terrain elevation and slope were strongly associated with the first axis. For the soil variables, 83.5% of the variance was explained by the first five axes, for which eight variables were above average: coarse fragments >2 mm, cation‐exchange capacity, soil pH, sand mass fraction, clay mass fraction and, silt mass fraction. In Table S2.2, additional details on ordination of this group can be found.

### 3.2 Habitat suitability model

**Figure 6:**
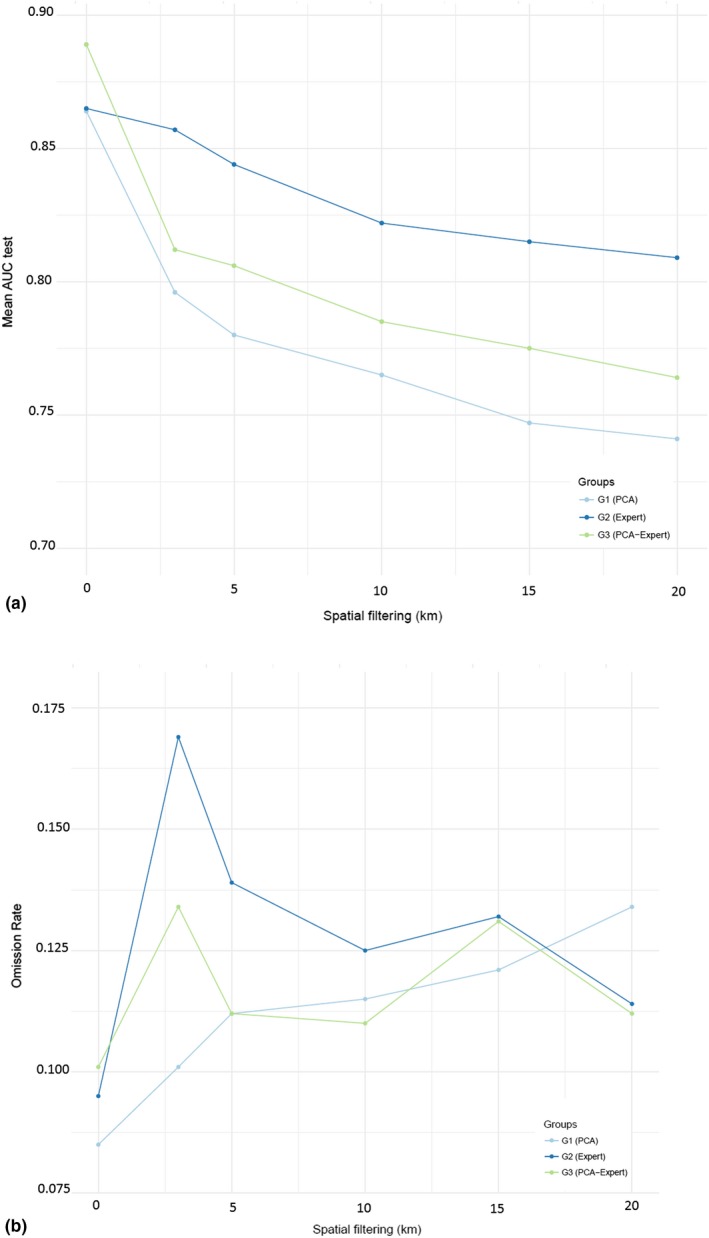
Results of the receiver operating characteristic (ROC) for the test data (AUC test) and omission rates (OR) in the 18 best models with lowest AICc (i.e., ΔAICc < 2) classified by group of predictors. (a) AUC and (b) OR. For each data‐partitioning approach, we adopted 10 interactions (*k* = 10)

Values of AUC and omission rates of the best 18 models with lowest AICc (i.e. ΔAICc < 2) are illustrated in Figure 6. All metrics are provided in Table S2.3.

#### 3.2.1 Maxent settings

There was a high degree of variation in both FC and *β* parameters among the best models. The most frequent regularization coefficient was *β* = 1.5 (44%), followed by *β* = 1 (39%) and *β* = 2.0 (17%). LQHPT feature classes appeared in 56% of the best models, including the final model.

#### 3.2.2 Choice of variables

PCA based on expert recommendations (Group 3) was the most appropriate method to select predictors on both unfiltered and filtered model based on the metrics (Table S2.3). Among unfiltered models, the highest AUC (0.89) was obtained for Group 3 and omission rates were within the expected errors (10%) (Figure 6a,b), but this model was considered unreliable due to overfitting caused by biased data used in calibration (Figure 6). The unfiltered model on the left showed a high probability of presence close to sampled occurrences (dark blue) and consequently, low out of sample (OOS) predictive power. This is not visualized on the filtered model on the right (at 10 km of tree distance), because its probability distribution is more regular.

Models based on all variables selected by experts (Group 2) had better discriminatory power than models based on the other groups, regardless of the scale of spatial filtering applied (3–20 km), with AUC values ranging from 0.80 to 0.86 (Figure 5a). However, these models had omission rates between 12% and 17%, that is, rates above the expected theoretical threshold (10%). This reflects low accuracy and predominance of false‐negative errors in the confusion matrix. However, after removing multi‐collinearity of the predictors selected by experts via PCA (Group 3), omission rates were reduced to 11%, as well as overfitting (Table S2.3). Therefore, this group of variables was selected for modeling the distribution of Amazon‐nut.

#### 3.2.3 Spatial filtering

**Figure 7:**
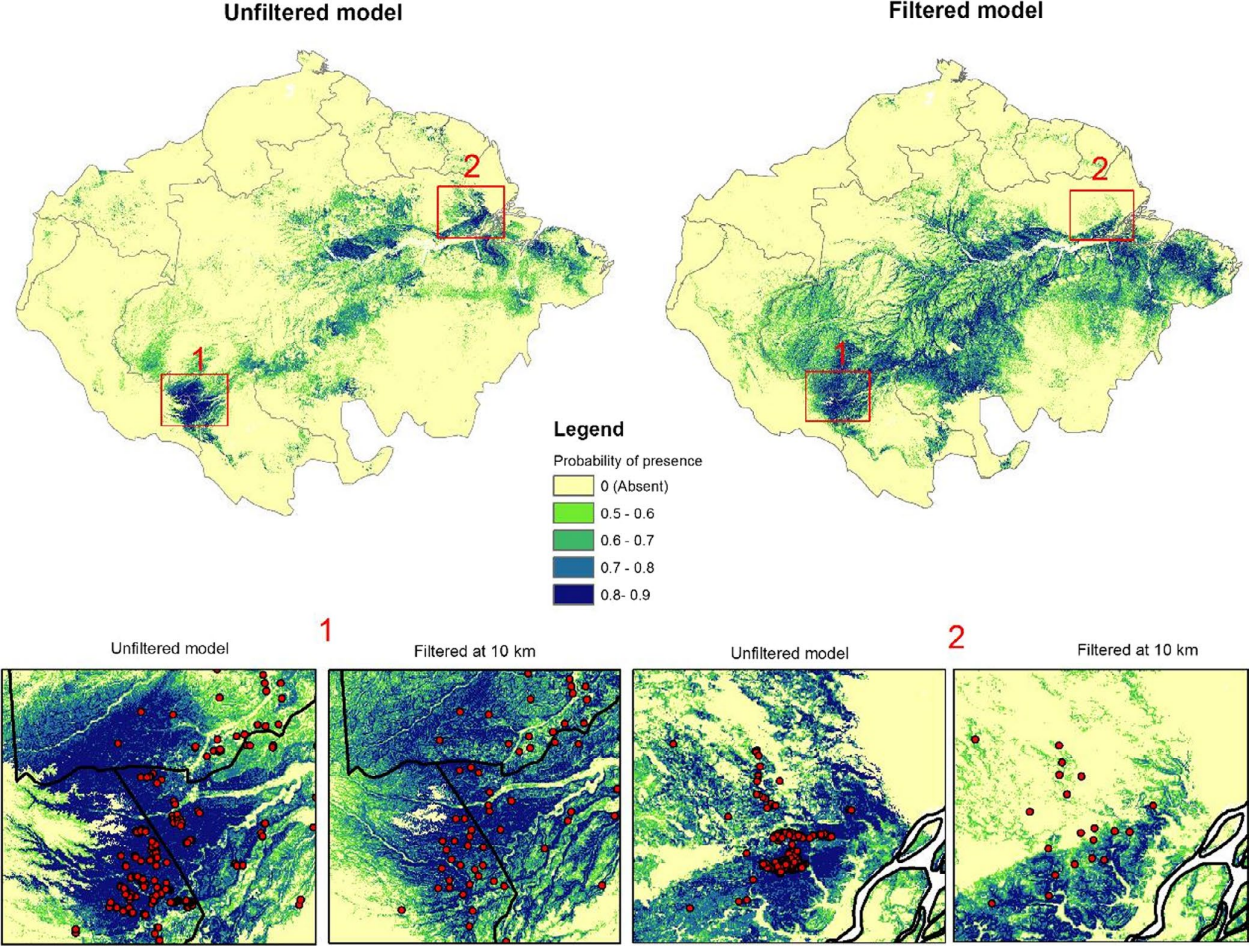
The best unfiltered and filtered models to estimate the Amazon‐nut (*B. excelsa*) habitat based on current environmental conditions (Group 3 of predictors). We highlighted for two areas where we had high density of sampled points (red points). Area1: at the border between Brazil, Peru and Bolivia. Area 2: southern Amapa State, Brazil

Unfiltered models were found to perform better than filtered models on the basis of AUC (Figure 6). However, through visual examination of the maps we noted strong signs of overfitting to training data for the former models (Figure 7), confirming that poorly fitted models with biased samples can have good discriminatory power (Lobo, Jiménez‐valverde, & Real, 2008), but may be nonetheless overfit. In Figure 7, the probabilistic maps suggested that overfitting was reduced with spatial filtering improving model quality. We highlighted two areas with high density of presence data in unfiltered and filtered models, and detected adjustment of biases and increase of the area extension predicted in the filtered model. However, our results expressed high similarity between filtered and unfiltered models using rarefied data from 3 to 20 km based on results of Schoener's D comparisons (Table S2.3).

The minimum distance between occurrence points was also highlighted through density curves. The unfiltered model showed signs of highly clustered data, featuring three peaks in the distribution curve, while in filtered models, curves were bell‐shaped with a single peak (Figure S2.1). The model simulated with 10 km of distance between records achieved a higher peak in the interval of 0.5–0.8 than other filtered models, as well as satisfactory discrimination power via AUC test (0.8) and lower omission rate (0.11).

**Figure 8:**
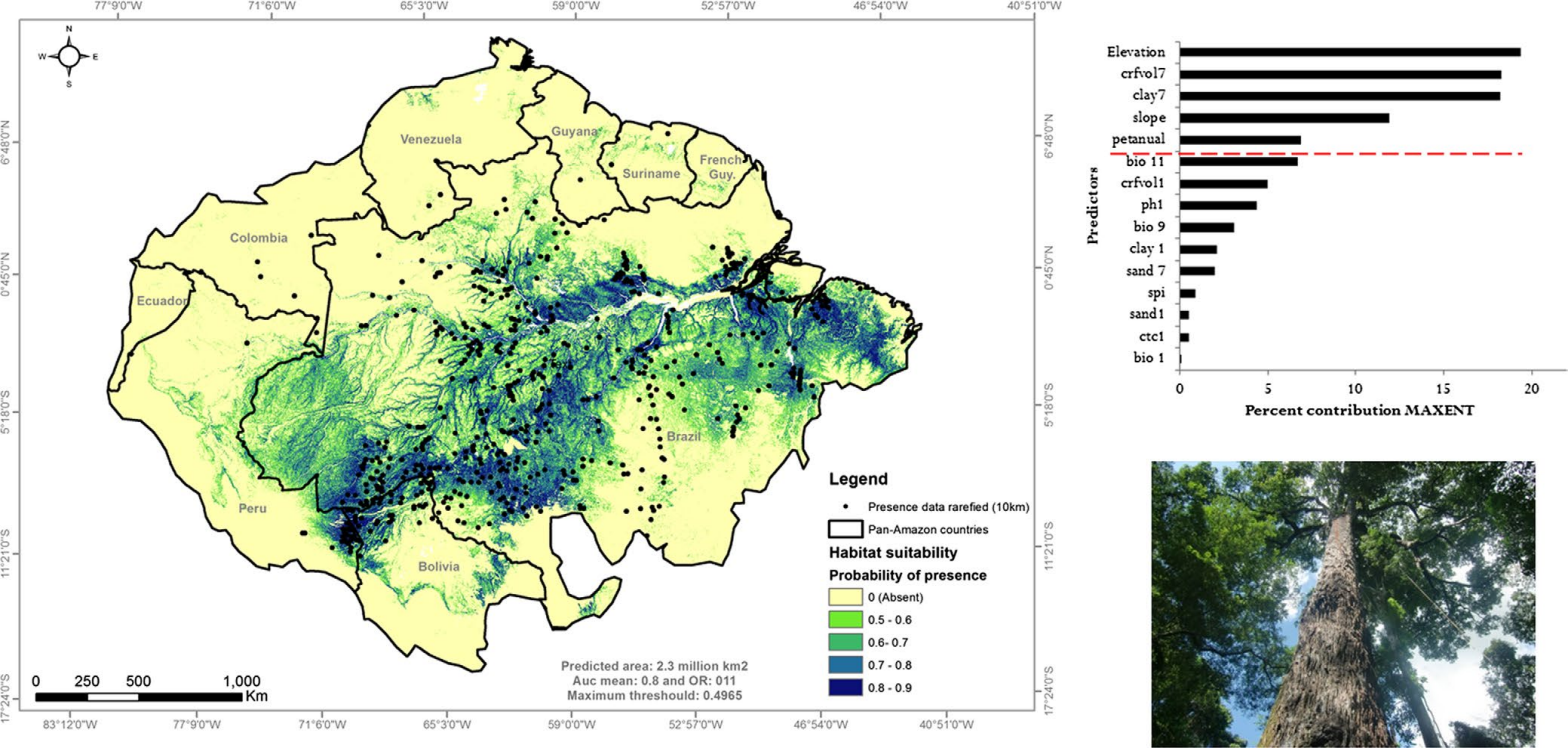
Distribution of suitable habitat for Amazon‐nut (*B. excelsa*) in the Pan‐Amazon to a probability of presence >0.5 (Max sss threshold) and percent of contribution of the variables in the final model. Dashed red line indicates five biggest contributions to Amazon‐nut distribution. Elevation (19.4%), coarse fragments volumetric >2 mm in % (18.3%), clay mass fraction % (18.2%), slope (11.9%), and annual potential evapotranspiration (6.9%)

The final model and the most important predictors are shown in Figure 8. This model was fit using records of Amazon‐nut distributed spatially filters at 10 km resolution (557 presence points), regularization multiplier (*β* = 1.5), feature classes combination (LQHPT), and Group 3 predictors. The minimum probability of occurrence was limited by the Max SSS threshold of 0.5, representing an omission rate of 11%. The five predictors with highest contribution highlighted by MAXENT were elevation (19.4%), coarse soil fragments >2 mm in % (18.3%), clay mass fraction % (18.2%), slope (11.9%), and annual potential evapotranspiration (6.9%). Our results suggest that under current environmental conditions, suitable habitat for Amazon‐nut is found across 2.3 million km^2^ or 32% of the Amazon Biome.

## 4 DISCUSSION

### 4.1 Amazon‐nut habitat suitability

We best model indicates across 2.3 million km^2^ is potentially suitable for *B. excelsa*. This area is far greater than that suggested by previous studies (1.3 million km^2^), in which the authors (Thomas et al., 2014), highlighted that some areas along the Tocantins River and in southeastern Amazonia may have been underrepresented. Our model identified that these and other areas in the eastern Amazon are suitable (Figure 8).

With respect to the most important predictors that control its spatial distribution, our results are similar to those found in other studies. In Peru, seed production was found to be positively correlated with clay content and negatively with sand content (Thomas et al., 2017). In Brazil, Guerreiro et al. (2017) found that the species has a preference soils with a clayey to very clayey texture. However, none of the previous studies identified the presence of coarse soil fragments >2 mm as being relevant to species distribution, despite its known occurrence in high stem densities on lateritic soils which contain coarse fragments (Müller, 1995; Salomão, 2009) and often are rich in iron oxide and aluminum (Horbe & Da Costa, 2005). Concerning chemical attributes, soil influence on fruit production has been shown to be positively associated with cation‐exchange capacity (Kainer, Wadt, & Staudhammer, 2007). However, other studies found highly productive trees in areas with higher levels of exchangeable Al and low soil pH, confirming that species can also be productive in acidic and less fertile soil (Costa, Tonini, & Filho, 2017).

Among topographic predictors, elevation was one of the strongest predictors. In the map, species probability of occurrence was lower at higher altitudes, as in the northern and southern extremes of the Amazon basin. Amazon‐nut trees have been recorded from sea level to ~400 m above sea level (ASL) (Thomas et al., 2014). Our data included specimens found up to 562 m ASL in the south of Para, Brazil. In addition, our model indicates that many lowland areas were suitable in contrast to the prevailing notion that this species prefers upland areas (Scoles et al., 2016). Indeed, species seed production has been shown to be lower when trees were close to rivers (Thomas et al., 2017). The unexpected inclusion of lowland areas as suitable Amazon‐nut habitat was discussed with experts.

Some experts emphasized that several islands in the Amazon estuary should not have been classified as suitable, because they are often flooded and have soils rich in silt, with growth condition adverse those where the species is commonly found. Others experts suggested that although Amazon‐nut occurrence in areas prone to flooding is rare, it can happen. They reported an example in the *lago capanã grande* reserve, in Manicoré, Amazonas, where the local community affirms that the Amazon‐nut trees in flooded areas are more productive than those found in nonflooded areas thanks to the presence of river sediments. Similarly, occurrences and high fruit productivity have been described in Peruvian Amazonian lowlands, notably in Madre de Dios (Nunes et al., 2012). Amazon‐nut population observed closer to the river has been recently associated with dispersal by ancient humans who strongly contributed to expand species distribution in the habitat (Thomas et al., 2014, 2015). For future studies, we recommend a more detailed investigation about Amazon‐nut suitability in periodically flooded areas using environmental data at finer spatial resolutions, also, taking into account frequency and duration of flooding.

Climate was less important than soil and topography to Amazon‐nut habitat suitability in the best model. This was unexpected given the known importance of climate to spatial patterns in floristic diversity across Amazonia (da Silva et al., 2011). However, our findings are similar to those of a recent study that highlighted the relative importance of edaphic conditions to plant occurrence in Amazon (Figueiredo et al., 2017). We attributed this result to recognized importance of soil attributes to Amazon‐nut ecology and productivity (Costa et al., 2017; Kainer et al., 2007). Moreover, we highlight that the percent contribution values ranked by MAXENT are determined by how much of variation a model with only that variable explains, it considers environmental variables separately (Bradie & Leung, 2017). A low variation in climate predictors was confirmed though our PCA analysis (Table S2.2).

Despite low variation, the annual potential evapotranspiration contributed with 7% in the final model. Derived from climatic variables, this predictor represents the amount of soil water lost by evaporation and transpiration from plants into the atmosphere under given conditions (Zomer, Trabucco, Straaten, & Bossio, 2006). Inclusion of this variable in the final model makes ecological sense as the Amazon‐nut is an emergent tree that receives a high level of solar radiation. Consequently, Amazon‐nut trees are vulnerable to drought and water loss. It has been noted that this species is most vulnerable to drought during the dry season, and that dry and warm conditions negatively affect species seed production (Thomas et al., 2017). Facing climate changes, forest loss and rapid land‐use changes, many uncertainties hover on Amazon‐nut future. Therefore, natural and human factors, as well as their consequences on the species distribution, must be urgently assessed to ensure its conservation.

The above reflections were supported by experts consulted who believe that the model was adequate to representing Amazon‐nut habitat suitability. Although, some areas were deemed underpredicted in Venezuela, Guyana, and Colombia. This was attributed to limited presence data obtained in these countries. In Brazil, the country that contains the greatest percentage of habitat for this species (91%), many microregions classified as suitable were confirmed by experts, such as: in Amazonas (microregion of Purus, Madeira, medium and low Rio Negro); southern of Amapá (microregion of Mazagao); Pará (microregion of Santarém, Óbidos, Itaituba, Tome‐Açú, Marabá); and Rondonia (Microregion of Porto velho). Experts also identified areas that were not suitable for *B. excelsa*, although the model identified them to be as, such as: in microregions of Roraima (Roraima) and Cruzeiro do Sul (Acre). According to these experts, this was not a commission error inherent to model because there are Amazon‐nut trees planted and growing in arboretums and nurseries, but not in natural forest in the Cruzeiro do Sul, for example. These potential areas not naturally occupied can be justified by ecological factors.

Unfortunately, biologic information was not considered in our model, due to the scarcity of spatial data. Fauna studies are often local and focused on population dynamic (demography, displacement, and food availability) (Haugaasen et al., 2012). Fauna habitat modeling may be extremely useful to tree distribution studies, but it is seldom investigated for terrestrial Amazon species. Besides fauna interaction, very little is known on Amazon‐nut dominated plants communities and their roles on species distribution.

### 4.2 Methodological aspects

Our results demonstrated that a hybrid strategy based on statistical modeling and expert opinion allowed identifying the best model for *B. excelsa*. This finding reinforces that the relationships among original predictors should be understood not only through their statistical behavior, but also by the ecological role they play in the species distribution. Although PCA is a highly informative ordination technique and has been extensively applied in community ecology since 1954 (Legendre & Legendre, 2012), interpretation of outputs requires biological knowledge (Janekovi & Novak, 2012).

Detailed biological knowledge is still scarce or incomplete for many if not most Amazonian plant species. Therefore, expert‐based information has been proposed as an alternative approach to identifying meaningful predictors in habitat modeling (Calixto‐Pérez et al., 2018). Our findings showed that PCA was effective in reducing omission error rates, data collinearity, and dimensionality, as well as preserving maximum variance, when applied to a set of variables preselected by experts. Thus, among 29 variables chosen by experts, fifteen were selected via PCA and used to fit our model. Five of them had a contribution of 75% in the best model found ensuring statistical and ecological representativeness.

Even using the best set of predictors, we observed that the discriminatory ability of filtered models measured by AUC was gradually reduced at larger filtering distances. Ironically, our most biased model (poorly fitted) received the highest AUC value. Similar results were found by Radosavljevic and Anderson (2014). This was expected, because the AUC has been shown to be insufficient for model evaluation when no true absence data are available (Jiménez‐valverde, 2012; Lobo et al., 2008). Through visual interpretation, we identified clear positive effects of spatial filtering on reducing overfitting (Figure 7), supporting previous research (Kramer‐Schadt et al., 2013). However, the challenge was to define at which distance the filtering became too strict, because statistically, there was high similarity between filtered models. We addressed this problem by comparing metrics and density curves (Table S2.4 and Figure S2.1).

The minimum distance of 10 km between presence data was considered appropriate to the adopted scale. Models using data filtered in this way displayed the highest peak in density curve in the interval of 0.5–0.8, satisfactory discrimination power via AUC test and lower omission rate. The same distance has been used and recommended in other studies of highly heterogeneous areas (Boria et al., 2014; Kramer‐Schadt et al., 2013). However, 10 km does not represent a distance between populations or groves, it was only chosen in order to reduce geographical bias existing in the data. If AUC would have been the only evaluation metric, it would have been misleading. But, together with other metrics and visual evaluation, this index was useful, because the biased models with highest AUC were used as reference to compare with other metrics.

Regarding Maxent settings, the variation of FC and *β* between experiments led us to conclude that these parameters should be fine‐tuned on a species and dataset‐specific basis (Radosavljevic & Anderson, 2014). However, contrary to our expectations, the data were well‐fitted to the combinations of all feature classes, usually suggested as default (Phillips & Dudík, 2008), when we compared to more simplified functions. Similar results were found by (Elith et al., 2011), when comparing models with all features to those using only the hinge function, with no differences in the predictive ability of either model were found. For the *β*, values ranged from 1 to 2 among the 18 best models. This corresponds with the optimal range obtained by Radosavljevic and Anderson (2014).

## 5 CONCLUSION

The outcomes suggest that we may be able to fit a robust habitat suitability model by developing a collaborative model‐building process. The combinations of statistical techniques with expert knowledge were decisive in the selection of predictors. The PCA despite being powerful should be complemented by ecological knowledge. By involving experts actively, we were also able to better define the addressed ecological and methodological questions, as well as to evaluate our results with their feedback. Other positive strategy for the construction of a reliable model was the application of spatial filtering that helped us identify the minimum distance between presence points. Fine‐tuning of model parameters also allowed us to understand their effects in the model quality. At last, combining qualitative and quantitative methodologies, we could identify spatial variations between models, evaluate metrics efficiency and the model accuracy.

The best model showed that 2.3 million km^2^ of the Amazon region is potentially suitable for *B. excelsa* based on the existence of appropriate environmental conditions. Topographic and soil variables were the predictors with the highest contribution to the model, expressing that geomorphology and soil physic are more important than soil chemistry and climate to explain species occurrence in the adopted scale (extension and grain size). It is also crucial to stress that the real habitat occupied by this species is smaller than 32% of the Amazon, mainly due to other ecological and anthropogenic factors, generally unknown or rarely monitored, such as predation, pollination, natural dispersal limitation, genetic variation, fragmentation, among others.

Our model can efficiently assist new site selections for planting; however, aggregation with additional information to reach planting success is strongly suggested, such as proximities with conserved forest fragments to allow pollination process; planting mixed with other species to facilitate the bee's flight up to the Amazon‐nut flowers; make seeds selection to ensure healthy individuals with high fruit productivity; and conduct management and forestry treatments. The generated model can also be used as basis for modeling studies considering future scenarios for climate change and to support conservation practices. We recommended that other studies be developed in a small scale, constantly including decision‐makers in the processes.

## CONFLICT OF INTEREST

None declared.

## AUTHORS CONTRIBUTION

Dr. TOURNE is the first author of this manuscript. She implemented the research design, analysis, results interpretation during her thesis, and wrote this manuscript. Dr. BALLESTER was Daiana's advisor during her PhD and contributed to this manuscript by advising, suggestion, technical support on geoanalysis and modeling, and manuscript writing. Dr. JAMES was Daiana's supervisor during her PhD and also contributed by advising on modeling and statistical analysis to fit models and manuscript reviewing. Dr. MARTORANO is an expert on modeling of climatic variables. She advised Daiana on variables selection, PCA analysis interpretation and helps to codesign a framework to improve Amazon‐nut habitat modeling, and manuscript reviewing. Dr. GUEDES is an expert on Amazon‐nut ecology. He shared his occurrence database and advised on research design, soil variables analysis and results interpretation found for this species, and manuscript reviewing. Dr. THOMAS is an expert on Amazon‐nut ecology and modeling. He shared his occurrence database and contributed by reviewing the used methodological steps and results interpretation found for this species.

## Data Availability

Amazon‐nut occurrence location presented here were collected from various data sources. Only Amazon‐nut occurrence data rarefied at a minimum distance at 10 km (557 points) from collectors and research projects have been made available to respect property rights; 10 km being the best distance to reduce bias for this database. It is listed in Table S1.3.
