# Supplementary material for: Strategies to optimize modeling habitat suitability of Bertholletia excelsa in the Pan‐Amazonia
Source: Ecol Evol. 2019 Oct 25;9(22):12623–38. doi: 10.1002/ece3.5726 (PMC6875584; doi:10.1002/ece3.5726)
Supplement: Supplementary file 1 [file ECE3-9-12623-s001.docx]

| **Number of occurence** | **state/country** | **source** |
| --- | --- | --- |
| 84 | PA/Brasil | First authors ’field survey |
| 24 | PA/Brasil | Plano de manejo Flota Trombetas |
| 74 | PA/Brasil | GIBF-MAPCAST - Lucieta Martorano/ Embrapa |
| 32 | PA/Brasil | CRIA-MAPCAST- Lucieta Martorano/ Embrapa |
| 5 | PA/Brasil | Dário do Amaral e Diego de Sousa  / Museu Goeldi/ IBAMA |
| 2 | PA/Brasil | REFLORA |
| 22 | PA/Brasil | LITERATURA |
| 20 | PA/Brasil | Maria Beatriz Ribeiro/INPA |
| 2 | PA/Brasil | Plano de manejo Flona Trairão |
| 16 | PA/Brasil | Herbário Embrapa |
| 11 | AC, AM, RR,RO,MT,PA | INPA-MAP;CAST |
| 39 | PA, RR, RO, AM, | LITERATURA-MAPCAST |
| 34 | AP/Brasil | Resex Rio Cajari - Marcelino Guedes/ Embrapa Amapá |
| 205 | AP/Brasil | Capoeira Marinho – Marcelino Guedes/Embrapa Amapá |
| 15 | AC, AM, MT,PA | Museu Goeldi - MapCast |
| 105 | AP/Brasil | Laranjal do Jari - Marcelino Guedes/Embrapa Amapá |
| 641 | AP/Brasil | Capoeira Marinho e Martins- Marcelino Guedes/Embrapa Amapá/Mapcast |
| 540 | PA/Brasil | Marcelino Guedes/Embrapa Amapa/Mapcast |
| 29 | PA/Brasil | Resex Lago Campana grande – Ricardo Scoles/UFOPA |
| 8 | PA/Brasil | Raquel dos Santos/USP |
| 14 | RR,AM,MT,PA,AM,AC | Katia da Silva/ Embrapa/Mapcast |
| 1078 | AM,PA, AP, AC, RO, RR, MT, Bolívia, Perú, Colombia, Venezuela, Guiana, Suriname | Evert Thomas database (CGIAR): Andes to Amazon Biodiversity program (Botanical Research Institute of Texas); Herbarium of The New York Botanical; Garden Rapid Assessment program (Conservation International); Royal Botanic Gardens, Kew; Missouri Botanical Garden; Sylvain JM Desmoulière database |

**SUPPORTING INFORMATION**

**S1: General information data and elementary soil data analysis.**

Table S1.1 *B.excelsa’s* occurrence records obtained from researchers’ databases, herbarium data, museums’ geographical data, scientific publications and field survey data from 2015 to 2017.


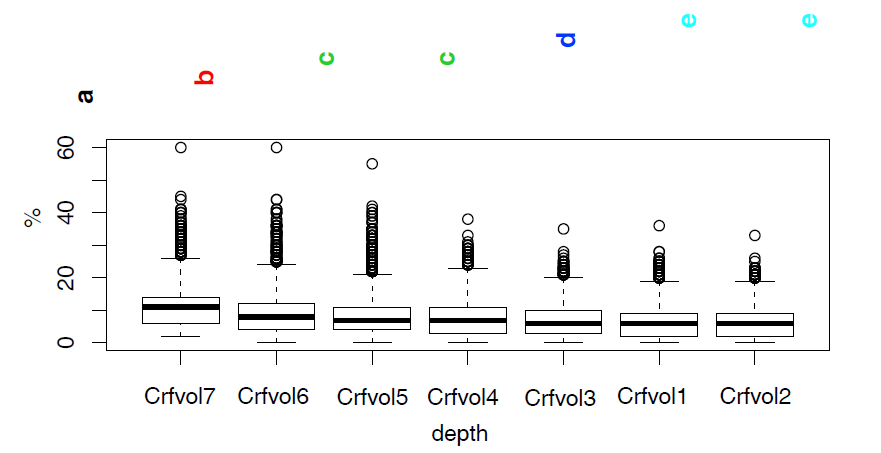

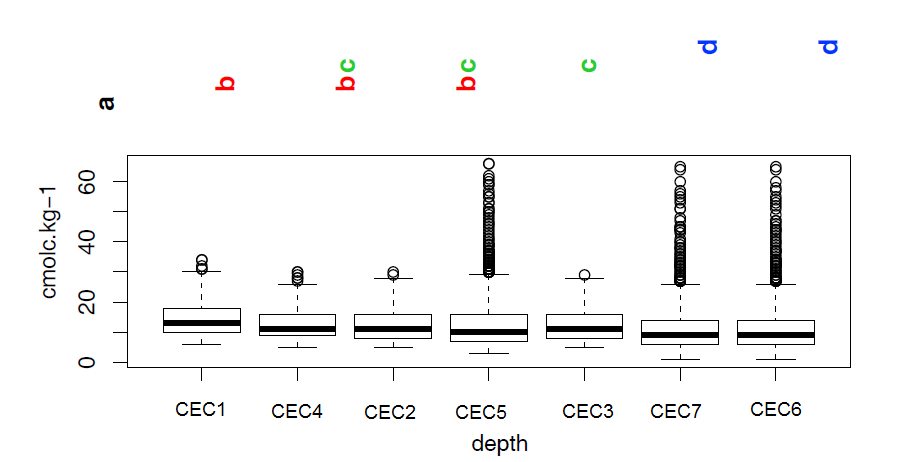

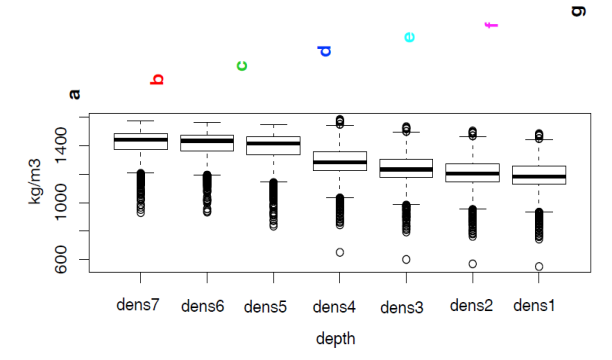

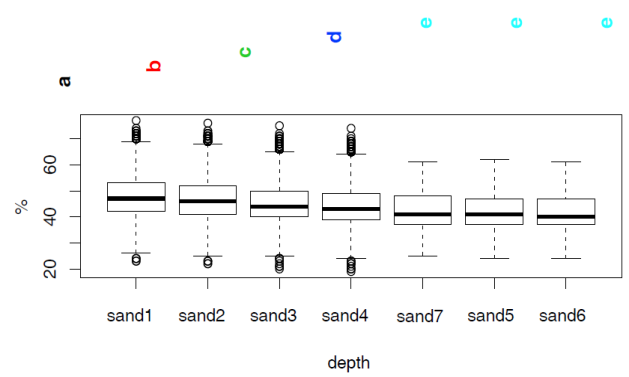

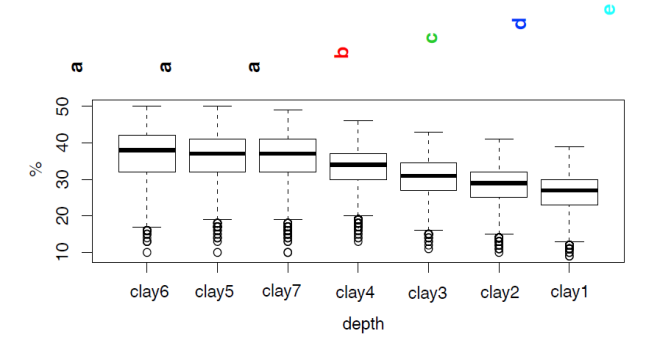

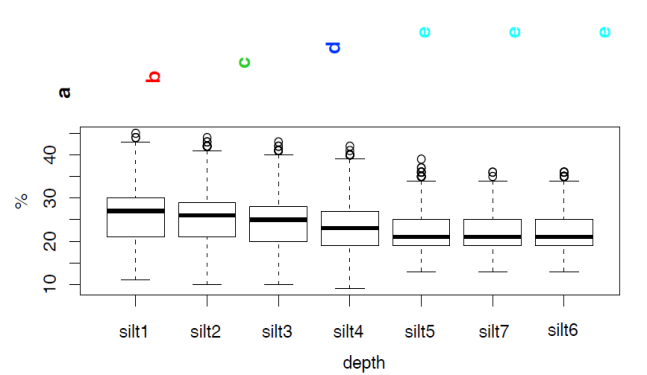

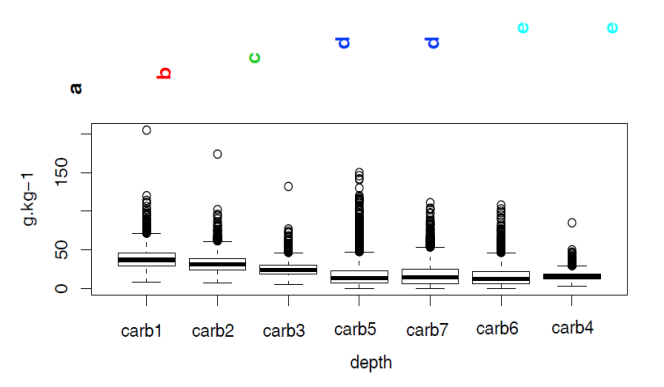

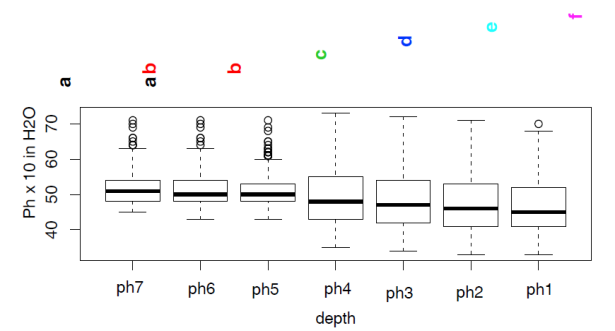


Figure S1.1 Boxplot graphics of soil variables submitted for pairwise mean comparisons in depths using *Tukey’s test* (HSD) implemented on *multcompBoxplot* R function. Numbers from 1 to 7 indicate the depth (0cm, 5cm, 15cm, 30cm, 60cm, 100cm and 200cm). The used variables were sand mass fraction % (sand), silt mass fraction % (silt), clay mass fraction % (clay), Cation Exchange Capacity in cmolc. kg^-1^ (CEC), Organic Carbon in g. kg^-1^ (carb), Bulk density (fine earth) in kg / m^3^ (dens), Soil pH x 10 in H2O (ph) and Coarse fragments volumetric in % (cfrvol). Differences between means in the depth that share a letter and color are not statistically significant (P< 0.05).

In the Amazon region, the soil proprieties with mean statistically significant in the following depth are: sand (1,2,3,4,7); clay (1,2,3,4,7); Silt (1,2,3,4,5); Carbon (1,2,3,5,6); Crfvol (1,3,5,6,7); CEC (1,3,4,7); dens (1,2,3,4,5,6,7) and PH (1,2,3,4,5,7). The relations between them were also analyzed in this study.

**S2: Summary of ordination data and similarity test between models.**

Table S2.1 Summary of PCA in the geographic space of the Amazon (**Group1**): Proportion of variance explained in the original data (1^st^PCA) and proportion of variance explained for only the most important variables in the principal axes (2^st^PCA). The accumulated variance with criteria (ɑ_0_ > 0.80) was adopted. Eigenvectors for the 2^nd^ PCA close to 1 indicate a strong relation between axes and predictors.

|  |  | **Principal components** | | | | |  |
| --- | --- | --- | --- | --- | --- | --- | --- |
|  |  | **PC1** | **PC2** | **PC3** | **PC4** | **PC5** |  |
| **1^st^PCA (%Variance accum.)** | Climate | 43.29 | 74.52 | **87.11** | - | - |  |
|  | Soil | 38.98 | 62.21 | 74.31 | **82.04** | - |  |
|  | Geo | 37.49 | 53.37 | 65.49 | 75.46 | **81.84** |  |
|  |  | **PC1** | **PC2** | **PC3** |  |  |  |
| **2^nd^PCA (%Variance accum.)** | Climate | 56.03 | **92.40** | - | - | - |  |
|  | Soil | 47.46 | 68.37 | **81.92** | - | - |  |
|  | Geo | 41.40 | 65.23 | **85.22** | - | - |  |
| **Predictors selected** | | **Eigenvectors (loading*sdev)** | | | |  |  |
| **Climate** | BIO9 | 0.93 | 0.27 |  |  |  |  |
|  | PETdrq | 0.81 | -0.54 |  |  |  |  |
|  | SWCdrq | 0.33 | 0.93 |  |  |  |  |
| **Soil** | dens1 | -0.51 | -0.38 | -0.72 | - | - |  |
|  | dens2 | -0.51 | -0.38 | -0.72 | - | - |  |
|  | dens3 | -0.51 | -0.38 | -0.72 | - | - |  |
|  | dens4 | -0.51 | -0.38 | -0.72 | - | - |  |
|  | ph1 | 0.65 | 0.46 | -0.51 | - | - |  |
|  | ph2 | 0.65 | 0.46 | -0.51 | - | - |  |
|  | ph3 | 0.65 | 0.46 | -0.51 | - | - |  |
|  | ph4 | 0.65 | 0.46 | -0.51 | - | - |  |
|  | silt1 | 0.81 | -0.48 | 0.12 | - | - |  |
|  | silt2 | 0.81 | -0.48 | 0.12 | - | - |  |
|  | silt3 | 0.81 | -0.48 | 0.13 | - | - |  |
|  | silt4 | 0.81 | -0.48 | 0.13 | - | - |  |
| **Geophysical** | asp | 0.03 | -0.04 | 1.00 | - | - |  |
|  | hvalbotton3 | 0.53 | -0.77 | -0.03 | - | - |  |
|  | aversoilsed6 | -0.92 | 0.20 | 0.02 | - | - |  |

**BIO9**: Mean Temperature of Driest Quarter ; **PETdrq**: Evapotranspiration of Driest Quarter ; **SWCdrq**: Soil water content of Driest Quarter; **dens1:** Bulk density (fine earth) in kg / m3 (0cm); **dens2:** Bulk density (fine earth) in kg / m3 (5cm); **dens3:** Bulk density (fine earth) in kg / m3 (15cm); **dens4:** Bulk density (fine earth) in kg / m3 (30cm); **ph1:** Soil pH x 10 in H2O (0); **ph2:** Soil pH x 10 in H2O (5cm); **ph3**: Soil pH x 10 in H2O (15cm); **ph4:** Soil pH x 10 in H2O (30cm); **silt1:** silt mass fraction %(0); **silt2:** silt mass fraction %(5cm); **silt3**: silt mass fraction %(15cm); **silt4:** silt mass fraction % (30cm); **asp:** Aspect; **hvalbotton3:** hill-slope_valley-bottom;**aversoilsed6:** average_soil_and_sedimentary-deposit_thickness.

Table S2.2 Summary of PCA calculated to set of variables selected by the experts (**Group3**): Proportion of variance explained to the original data and the most important variables in the principal axes. The accumulated variance with criteria (ɑ_0_ > 0.80) was adopted. Eigenvectors next to 1 indicate a strong relation between axes and predictors.

|  |  |  |  |  |  |  |  |
| --- | --- | --- | --- | --- | --- | --- | --- |
|  |  | **PC1** | **PC2** | **PC3** | **PC4** | **PC5** |  |
| **%Variance accum.** | Climate | 53.6 | **86.8** | - | - | - |  |
|  | Soil | 38.2 | 62.4 | 72.8 | 79.3 | **83.5** |  |
|  | Geo | 68.5 | **90.0** | - | - | - |  |
| **Predictors selected** | | **Eigenvectors (loading*sdev)** | | | |  |  |
| **Climate** | BIO1 | 0.84 | -0.50 | - | - | - |  |
|  | BIO9 | 0.88 | -0.40 | - | - | - |  |
|  | BIO11 | 0.85 | -0.49 | - | - | - |  |
|  | PETa | 0.35 | -0.88 | - | - | - |  |
| **Soil** | Crfvol1 | 0.78 | 0.02 | 0.32 | 0.43 | 0.12 |  |
|  | Crfvol7 | 0.76 | 0.04 | 0.29 | 0.42 | 0.11 |  |
|  | CEC1 | 0.76 | -0.34 | -0.05 | -0.38 | -0.07 |  |
|  | ph1 | 0.63 | -0.21 | 0.47 | -0.49 | -0.06 |  |
|  | sand1 | -0.33 | 0.81 | 0.32 | -0.21 | 0.28 |  |
|  | sand7 | -0.23 | 0.84 | 0.05 | 0.11 | -0.31 |  |
|  | clay1 | -0.13 | -0.77 | -0.21 | 0.02 | -0.47 |  |
|  | clay7 | -0.27 | -0.85 | -0.08 | -0.01 | 0.34 |  |
|  | silt7 | 0.79 | -0.03 | 0.05 | -0.21 | -0.05 |  |
| **Geophysical** | elev | -0.86 | 0.35 | - | - | - |  |
|  | spi | 0.72 | 0.69 | - | - | - |  |
|  | slop | -0.89 | 0.22 | - | - | - |  |

**BIO1:** Annual Mean Temperature; **BIO9:** Mean Temperature of Driest Quarter; **BIO11:** Mean Temperature of Coldest Quarter; **PETa:** Annual Potential evapotranspiration; **Crfvol1:** Coarse fragments volumetric >2mm in % (0); **Crfvol7:** Coarse fragments volumetric >2mm in % (200cm); **CEC1:** Cation Exchange Capacity in cmolc. kg-1 (0); **ph1:** Soil pH x 10 in H2O (0); **sand1:** sand mass fraction % (0);**sand7:** sand mass fraction % (200cm); **clay1:** clay mass fraction % (0); **silt1:** silt mass fraction %(0) **elev:** terrain elevation model;**spi:** Stream Power Index;**slop:** slope

Table S2.3 Pair-wise Schoener’s D statistic measuring similarity between filtered models from group 3. The statistic ranges from 0 (no overlap) to 1 (the distributions are identical).

|  | **Unfiltered** | **Filtered at 3km** | **Filtered at 5km** | **Filtered at 10km** | **Filtered at 15km** | **Filtered at 20km** |
| --- | --- | --- | --- | --- | --- | --- |
| **Unfiltered** | 1 |  |  |  |  |  |
| **Filtered at 3km** | 0.91 | 1 |  |  |  |  |
| **Filtered at 5km** | 0.89 | 0.91 | 1 |  |  |  |
| **Filtered at 10km** | 0.86 | 0.89 | 0.94 | 1 |  |  |
| **Filtered at 15km** | 0.85 | 0.88 | 0.92 | 0.94 | 1 |  |
| **Filtered at 20km** | 0.85 | 0.88 | 0.93 | 0.96 | 0.95 | 1 |

Table S2.4 Metric calculated for the 18 best models with lowest AICc (i.e. ΔAICc < 2).

| **Unfiltered models** | **Preditors groups** | **Presence data (Filtre at km)** | **Number of points** | **FC** | **RM** | **AUCTEST** | **OR10** | **ΔAICc** |
| --- | --- | --- | --- | --- | --- | --- | --- | --- |
|  | 1 | Unfiltered | 3252 | LQHP | 1 | 0.864 | 0.09 | 0.00 |
|  | 2 |  |  | LQP | 1 | 0.865 | 0.10 | 0.00 |
|  | **3** |  |  | **T** | **2** | **0.889** | **0.10** | **0.00** |
| **Filtered models** | 1 | 3 | 759 | LQHPT | 1 | 0.796 | 0.10 | 0.00 |
|  |  | 5 | 673 | T | 1 | 0.78 | 0.11 | 0.00 |
|  |  | 10 | 557 | T | 1 | 0.765 | 0.12 | 0.00 |
|  |  | 15 | 495 | T | 1.5 | 0.747 | 0.12 | 0.00 |
|  |  | 20 | 445 | T | 1.5 | 0.741 | 0.13 | 0.00 |
|  | 2 | 3 | 759 | LQHPT | 1 | 0.857 | 0.17 | 0.00 |
|  |  | 5 | 673 | LQHPT | 1.5 | 0.844 | 0.14 | 0.00 |
|  |  | 10 | 557 | LQHPT | 1.5 | 0.822 | 0.13 | 0.00 |
|  |  | 15 | 495 | LQHPT | 1.5 | 0.815 | 0.13 | 0.00 |
|  |  | 20 | 445 | LQHPT | 2 | 0.809 | 0.11 | 0.00 |
|  | **3** | 3 | 759 | T | 1 | 0.812 | 0.13 | 0.00 |
|  |  | 5 | 673 | LQHPT | 1.5 | 0.806 | 0.11 | 0.00 |
|  |  | **10** | **557** | **LQHPT** | **1.5** | **0.785** | **0.11** | **0.00** |
|  |  | 15 | 495 | LQHPT | 2 | 0.775 | 0.13 | 0.00 |
|  |  | 20 | 445 | LQHPT | 1.5 | 0.764 | 0.11 | 0.00 |


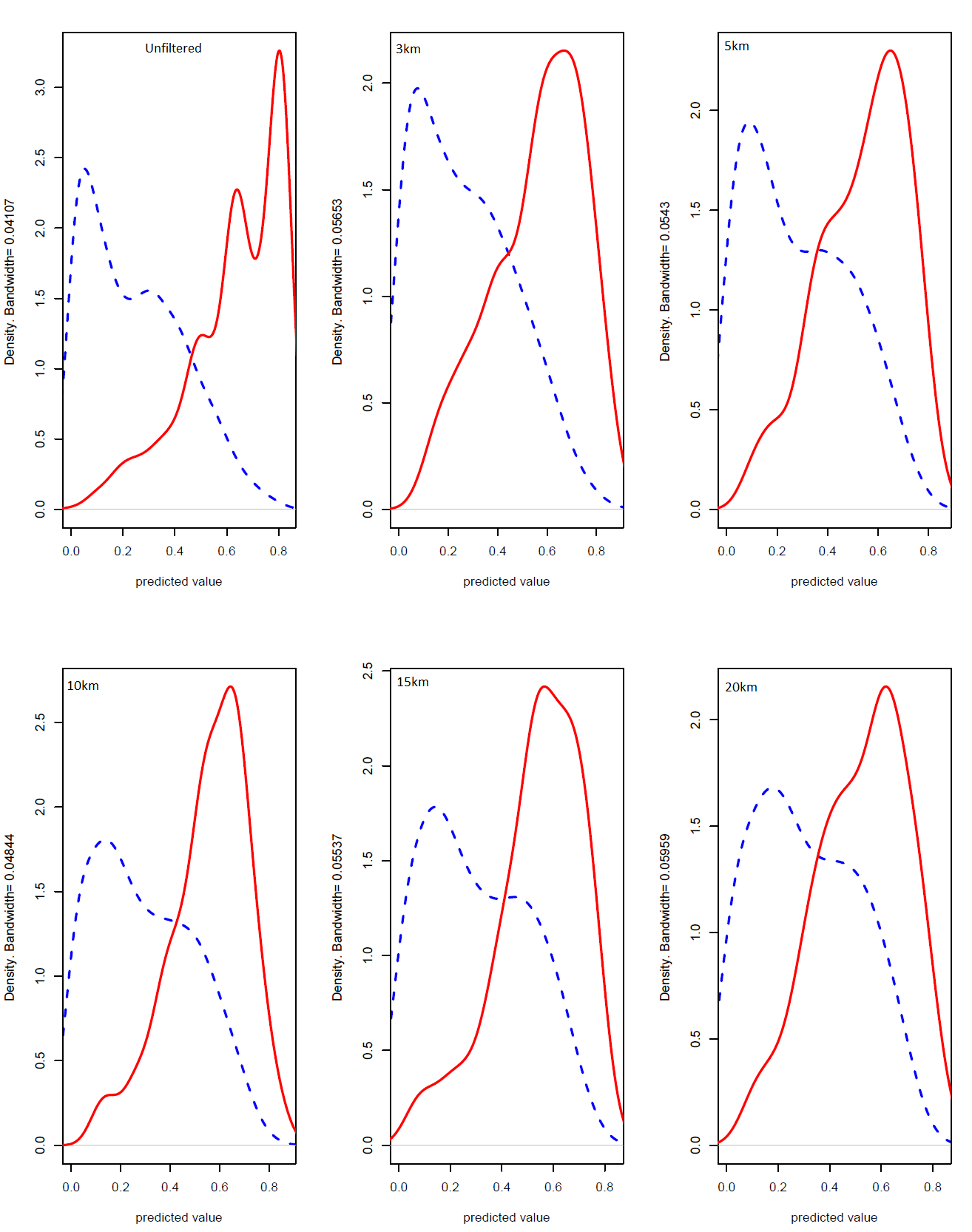


Figure S2.1 Density curves estimated in the best unfiltered and filtered models from group 3. The proportion of presence data are in red and absence data (background data) are represented by a blue dashed line.
